# Heart-Clots and Embolism, and Esmarch’s Bandage in Amputation

**Published:** 1876-05

**Authors:** John P. Wall

**Affiliations:** Tampa, Fla.


### HEART-CLOTS AND EMBOLISM, AND ESMARCH'S BANDAGE IN AMPUTATION.

By JOHN P. WALL, M.D., of Tampa, Fla.

As the following cases may not be devoid of interest, they are offered for publication.

Lizzie, H., colored, about forty years old, was delivered of a healthy male child October 12th, 1875. She was the mother of several children, the one before the last being about eleven years of age. She was naturally stout, and had grown corpulent. Convalescence from the puerperal state was apparently prompt and without accident. I was called to see her Nov. 29th, and found her complaining of pain in the right hypochondrium, extending sometimes to the praecordia, and becoming very severe at this latter point. She said that on lying down she suffered with great difficulty in breathing, and in consequence she was unable to sleep at night. The pulse was small, weak, and rather frequent, but without irregularity or intermittence; temperature normal. The sounds of the heart were weak and its impulse feeble, but on account of her obesity, with very large breasts—affording ample nourishment to the infant—I did not know but that the sounds and the impulse might be due to these conditions. The difficulty in respiration in the recumbent position made me suspect, however, that there was some serious heart trouble, though not arriving at a position conclusive as to its nature.

From the patient I learned that these symptoms had commenced about a week previous to my visit, and that she dated their inception from an angry scuffle which she had had with a small negro man in ejecting him from her house.

Not fully understanding the nature of the case, the treatment adopted was rather expectant and temporizing. The appearance of the tongue and condition of the bowels indicating a purgative, one was prescribed, with sinapism over seat of pain. No marked benefit being derived after several days, and the greatest pain being located in the right hypochondrium, a blister was applied to the latter region with considerable relief from the pain on the next day. On the third day after the application of the blister I was hurriedly called to see her, as she was said to be dying; this was December 6th. As she resided in the suburbs of town, it was not long before I saw her. Found her supported in a chair, and learned that in walking across the room she had suddenly fallen. There was no coma, and the eyes were open and naturally winking, but no response could be got to any question asked her. At the first blush I thought there must be considerable hysteria mixed up in the case as it then presented itself. The pulse, however, was very small and weak, and increased in rapidity, while the extremities grew cool and perspiration about the head became profuse. Ordered some whisky to be given, and left for an hour. On my return was informed that she could not be got to swallow the stimulant, and found the coldness of the extremities increased. Gave directions for the administration of the stimulant by enemata, and to be repeated every hour or two.

By this time I had pretty well determined in my own mind that these symptoms indicated cerebral embolism. Late on the same afternoon Drs. Branch and Benjamin saw the case with me, but neither of these gentlemen seemed to have any clear idea of its character, though both agreeing in a speedy unfavorable prognosis, as the condition of the patient still remained about the same as previously described. The use of stimulant enemata was ordered continued during the night at intervals of two hours. Having to visit a patient some distance in the country the following day, I saw the patient about daylight, and found that she had fairly reacted, though pulse still weak and rapid. She was still aphasic and could not swallow; no paralysis noticed. Ordered nutrient enemata, and requested Dr. H. R. Benjamin to notice the case during my absence, which he kindly did, but without making any change.

When I saw the patient the following morning, third day of the sudden attack, she could swallow and say "yes" and "no." The left cheek was slightly paralyzed, and there was considerable paresis in both right extremities, as indicated by a dragging of the right foot in walking, and an indisposition to use the right hand. The tongue was not deflected from a straight line on protrusion, though considerable dribbling of saliva; no disparity in size of pupils nor any strabismus, ptosis, cophosis, nor anæsthesia.

For the first few days she appeared to improve, and was soon able to sit up and walk about the room. She could utter a few more words, such as "Come here, Doctor; O, Lord!" but beyond these all efforts to talk were a senseless jabber. By her manner and action she seemed quite anxious about herself, and though usually restless and disposed to jabber, would keep still and quiet for short periods when told to do so for purposes of examination. Without being able to describe it, it was manifest that there was lacking that mental equilibrium of a sound intellect; and yet, from the impossibility of judging of the mental condition of one incapacitated for expression in speech, it becomes exceedingly difficult, if not wholly impossible, to determine the amount of mental aberration present in such cases. I may here state that she was able to recognize, in most cases, those who visited her, and sometimes called their names.

In the course of a week the respiration, which had all along been more rapid than normal, became somewhat laborious, with working of the nostrils. There was marked dulness on the right side, with slight bronchial breathing posteriorly over the region of the scapula.

At this time—about the seventh day after the sudden attack—Mr. John Wilcox,* a distinguished and most accomplished physician, from Greencastle, Indiana, saw the case with me. On a close examination, besides the dulness of the right side, there was dulness in the left hypochondrium, indicating enlargement of the spleen. Pulse weak and a little upwards of 100; temperature 100 F. Dr. W. thought there was pneumonia of right lung and enlargement of the spleen, and suggested a prescription with carbonate of ammonia as the basis, which was ordered, though the patient could be induced to take but little of it. Next day those in attendance said she had spit a little blood, but at no time did I discover anything in the sputa characteristic of pneumonia. All along there had been great deficiency in the normal amount of sleep, and now at night the patient became so noisy as to prevent any of the other inmates from sleeping. I tried both bromide of potassium and chloral hydrate, with the effect of aggravating the sleeplessness. With morphine she rested better, and I finally settled down to a grain each of opium and digitalis, to be given *pro re nata.*

Thus the case remained in *statu quo*, gradually losing flesh, however, until the latter part of December, when the two right extremities began to swell, that of the leg becoming so great

---

*Dr. Wilcox was here on account of his health, and after great suffering with ulcerative pharyngitis, died February 1st, 1876. His professional ability, which was truly great, was equaled by the private virtues of the man. Religious in his life, he exemplified in his slow-approaching death the beauty and consolation of a full trust in our Redeemer's atonement.

as to confine her to bed; the arm never very badly. Finally the skin gave way on the leg, leading to ulceration and a constant serous drain, sufficient to keep her bed continually wet. The patient slowly and gradually grew worse, and died on the 19th of January. For a week before death she passed her excrements in bed, though throughout, so far as I could learn, there had been no trouble either in retaining or voiding the contents of the bladder. The bowels were moved for the most part with enemata. As I had never seen a similar case, I watched it with great interest from the first, and after some trouble succeeded in being permitted to make an autopsy, twenty-four hours after death.

Dr. H. P. Benjamin was present, but on account of having some abrasions on the hands, was unable to give me much assistance in the dissection. The brain was first examined. On incising the scalp from ear to ear over the vertex, it was found much thickened with adipose deposit, and a spot of ecchymosis, two inches in diameter, was observed at the posterior termination of the sagittal suture, for which there was no assignable cause in the way of a blow or a fall. The scalp being turned down anteriorly and posteriorly, the skull-cap was removed in the usual manner with saw and chisel. The brain was then carefully removed. It seemed of normal weight and size, while its surface presented no unusual appearances. Laid on its base, it was carefully sliced horizontally in thin sections with an amputating knife, and was found of the ordinary consistence, with slight darkish pigmentation of the white matter. Nothing unusual was seen until the sections reached near the corpus callosum, bringing into view the anterior cerebral arteries. The left was more rigid and of a lighter color than its fellow, and on being seized and drawn on, it and its branches were easily dragged out of a softened spot near the base of the brain and just porterior to the fissure of Sylvius, the last section having exposed the summit of the softening. The *ramolissement* was about half an inch in depth, and something over an inch in diameter, though not extending to the surface either at the side or base of the brain. The softened cerebral tissue was of the consistence of thick pus, and was readily washed away by a small stream of water. The trunk of the left anterior cerebral artery contained a continuous whitish clot, portions of which were readily pressed out on its section. The initial point of primary occlusion was not discovered.

The thorax was next opened in the usual manner by division of the costal cartilages and removal of the sternum. The right pleural cavity was nearly filled with a redish serus effusion, compressing the lung backwards. The pericardium was also distended with a similar effusion. The heart was now removed; it was large and flabby. White fibrinous clots, apparently undergoing fatty degeneration, were found in both sides of the heart, extending through the auriculo-ventricular valves into the respective auricle and ventricle of each side. On the right side the clot was transfixed by two of the chordæ tendinæ of the tricuspid valve; on the left side it was transfixed in a similar manner by one of the chordæ tendinæ of the mitral valve, thus allowing the two extremities of the two respective clots to play loosly in the auricle and ventricle of the two sides of the heart. The right clot was between two and three inches in length, the left not quite so long. Each clot presented the appearance somewhat of a cylinder of wheaten dough flattened by pressure, with some constriction where they passed through the auriculo-ventricular openings. That of the left side was the most constricted, while that on the right side abridged considerably the orifice between the auricle and ventricle, barely permitting the introduction of the finger. In thickness each clot was about one-fourth of an inch, and in breadth near an inch, except at the constricted points. In the right ventricle similar smaller clots were also found, with one extremity as if clasped to the ventricular wall by the columnæ carnæ. All these clots were of a milkish white appearance, somewhat granular and friable, with an unctuous feel when small fragments were crushed between the fingers. Oil globules and granular matter were discoverable under the microscope.

The wall of the right ventricle, near its base, had also undergone fatty degeneration, being yellowish, friable, and easily torn. The lungs were not thoroughly examined, though it was ascertained that the larger branches of the right pulmonary artery were filled with continuous whitish clots, similar to what was found in the left anterior cerebral. In the right lung there were a few points of lobular infarction, on the pleural surfaces of which there was a slight deposit of lymph. The lung, however, was free from general pneumonic disease, and, though some heavier than the left, was for the most part crepitant and inflatable.

The liver and spleen were next examined. The spleen was found healthy and of the normal size, much to my disappointment, for on account of the dulness in the region of the spleen I expected to find it enlarged, with embolism of the splenic artery. The liver, however, was very large, extending well over to the left, and was the occasion of the dulness in that region. Its surface presented a yellowish hue, and its parenchyma was of the same color, soft and friable, indicating fatty degeneration.

At this point I was compelled to bring my dissections to an end, and consequently did not determine whether or not there was embolism also of the hepatic artery, which I believe to have been the case. It would also have been interesting to have looked for arterial embolism of the main trunks of the two right extremities had I had time for so extended dissections.

*Remarks.*—It is hardly probable that the puerperal state had any direct etiological relation, save that possibly resulting from hyperinosis, toward the production of the cardiac thrombosis, which, no doubt, was the first link in the chain of subsequent pathological sequences.

"The conditions involved in the formation of heart-clot are: first, over-distension of the cavities of the heart from an undue accumulation of blood; second, an excess of fibrin in the blood or hyperinosis. A third condition may perhaps be added, namely, an abnormal coagulability of the fibrin, a condition to which the name *inopexia* has lately been applied. The accumulation of blood occasions a certain amount of paralysis from distension; hence the contents of the cavities are but partially expelled by the contraction of the muscular walls. There is more or less stagnation in the cavities. Under these circumstances the fibrin may coagulate, and it is isolated by the coagulation taking place slowly, and by the action of the cords and muscles, especially if the fibrinous constituent of the blood be in morbid excess. * * *

"When the heart-clot or thrombosis is in the cavities of the left side of the heart, there is generally weakness of the walls of the left ventricle and auricle, from either dilatation or fatty degeneration, the over-accumulation and stagnation being dependent on the loss of muscular power incident to these lesions."—*Flint.*

There can be no doubt but that the heart-clots in this case were formed long anterior to the time of dissolution, and from them came the fragments producing embolism in the cerebral and pulmonary arteries, and possibly also embolism in the hepatic and the branch arteries of the two right extremities. The effusion into the right pleural cavity is a condition, according to Juergensen, usually attending pulmonary embolism and infarction. On this point the reader is referred to volume v., p. 245, *et sequitur*, of Ziemssen's Cyclopedia of Medicine.

### ESMARCH'S BANDAGE AND RUBBER CORD IN AMPUTATION.

Seldom has any new method in surgical procedures so readily commended itself to the confidence of the profession as Esmarch's. The idea of relieving the condemned member of blood before proceeding to operate is by no means a new one, for I remember distinctly seeing my friend and former associate, Dr. E. M. Seabrook, resorting to elevation of the limb for this purpose in 1862, at Chimberazzo Hospital, Richmond, Virginia; nor did he put any claim to the idea as being original.

In all operations on the extremities of any importance, where it is desirable to restrain hemorrhage, it is *par excellence* the thing. It is also of special advantage to the isolated surgeon, who is often dependent on non-professionals for assistance, or half-handed doctors, but little better qualified.

On the 17th of last February, I was called to see a man thirty miles distant, who on the day before was accidentally shot in the left leg just above the ankle. The gun was loaded with the largest size of shot, known as buck-shot, and at the time of the discharge the muzzle was within a few feet of the recipient. The charge entered as a whole a little above the outer maleolus, and made its exit on the inner and anterior aspect of the leg, just above the instep, comminuting badly the lower ends of both bones of the leg. In fact, the comminution of the tibia extended several inches up the bone above the wound, thus precluding the possibility of a resection with any hope of retaining a useful foot.

The patient was forty-six years of age, in fair health and of good habits. As yet no inflammation had set up, and amputation was decided upon. Drs. Powledge and Beddingfield kindly assisted me; the former not in regular practice, a graduate of Atlanta in 1860, and the latter a recent graduate of Savannah.

Dr. Beddingfield administered the chloroform, while Dr. P. was to assist in the operation. As the patient got under the anæsthetic, the elastic bandage was applied to the leg, beginning at the toes and carrying it half way up the thigh. The limb was then encircled at the termination of the bandage with two turns of the rubber cord; the latter was put on the full stretch and tied. Never having seen used or used this apparatus before, I took the precaution to adjust also loosely, not tightening it at all, a tourniquet. The bandage was removed and the operation, five inches above the ankle, was performed by the usual circular method. In severing the limb but little blood was lost, all agreeing that it hardly amounted to an ounce. Only one or two of the arteries could be sufficiently distinguished to be taken up, and consequently the cord was removed, intending to control any hemorrhage with pressure or the tourniquet. But the blood did not rush back into the limb so rapidly as I expected, and when it did the extremities of the arteries could be seen pulsating without hemorrhage for several moments, sufficiently long to allow of their being taken up before the temporary contraction, occasioned by the sponging and atmospheric contact, yielded before the returning currents. After being assured that there were no small arteries left untied which might give occasion to troublesome hemorrhage when the divided structures recovered their normal temperature, lost by exposure, etc., the circular skin-flap was brought together with sutures and reinforced with short strips of plaster. The stump was dressed by applying a piece of old linen folded over the stump, and then bandaging the latter in a close-fitting manner, hardly amounting to tightness. No applications of cold water or anything else made to the stump. Morphine administered when necessary, which was once or twice.

The morning after the amputation there was no oozing, and the bandage had not even become stained on the surface. I suggested that three or four days be allowed to elapse before removing the first dressing, unless there were clear indications for earlier interference. The patient was left in the care of Dr. Powledge, and went on to recovery without accident.

As regards oozing, I am inclined to the opinion that where hyperæmia precedent to inflammation, or the latter exists, there will be oozing following operations; hence, intermediate amputations are notorious for this. It is also probable that if the wound or stump is too hastily dressed after the use of Esmarch's apparatus, oozing will follow. On these points further experience, however, is required.

